# Incidence of Revision Surgery After Decompression With vs Without Fusion Among Patients With Degenerative Lumbar Spinal Stenosis

**DOI:** 10.1001/jamanetworkopen.2022.23803

**Published:** 2022-07-26

**Authors:** Nils H. Ulrich, Jakob M. Burgstaller, Fabio Valeri, Giuseppe Pichierri, Michael Betz, Tamas F. Fekete, Maria M. Wertli, François Porchet, Johann Steurer, Mazda Farshad

**Affiliations:** 1 University Spine Centre Zurich, University Hospital Balgrist, University of Zurich, Zurich, Switzerland; 2 Horten Centre for Patient Oriented Research and Knowledge Transfer, University of Zurich, Zurich, Switzerland; 3 Institute of Primary Care, University and University Hospital Zurich, Zurich, Switzerland; 4 Department of Orthopedics and Neurosurgery, Spine Center, Schulthess Clinic, Zurich, Switzerland; 5 Division of General Internal Medicine, Bern University Hospital, Bern University, Bern, Switzerland

## Abstract

**Importance:**

Only limited data derived from large prospective cohort studies exist on the incidence of revision surgery among patients who undergo operations for degenerative lumbar spinal stenosis (DLSS).

**Objective:**

To assess the cumulative incidence of revision surgery after 2 types of index operations—decompression alone or decompression with fusion—among patients with DLSS.

**Design, Setting, and Participants:**

This cohort study analyzed data from a multicenter, prospective cohort study, the Lumbar Stenosis Outcome Study, which included patients aged 50 years or older with DLSS at 8 spine surgery and rheumatology units in Switzerland between December 2010 and December 2015. The follow-up period was 3 years. Data for this study were analyzed between October and November 2021.

**Exposures:**

All patients underwent either decompression surgery alone or decompression with fusion surgery for DLSS.

**Main Outcomes and Measures:**

The primary outcome was the cumulative incidence of revision operations. Secondary outcomes included changes in the following patient-reported outcome measures: Spinal Stenosis Measure (SSM) symptom severity (higher scores indicate more pain) and physical function (higher scores indicate more disability) subscale scores and the EuroQol Health-Related Quality of Life 5-Dimension 3-Level questionnaire (EQ-5D-3L) summary index score (lower scores indicate worse quality of life).

**Results:**

A total of 328 patients (165 [50.3%] men; median age, 73.0 years [IQR, 66.0-78.0 years]) were included in the analysis. Of these, 256 (78.0%) underwent decompression alone and 72 (22.0%) underwent decompression with fusion. The cumulative incidence of revisions after 3 years of follow-up was 11.3% (95% CI, 7.4%-15.1%) for the decompression alone group and 13.9% (95% CI, 5.5%-21.5%) for the fusion group (log-rank *P* = .60). There was no significant difference in the need for revision between the 2 groups over time (unadjusted absolute risk difference, 2.6% [95% CI, −6.3% to 11.4%]; adjusted absolute risk difference, 3.9% [95% CI, −5.2% to 17.0%]; adjusted hazard ratio, 1.40 [95% CI, 0.63-3.13]). The number of revisions was significantly associated with higher SSM symptom severity scores (β, 0.171; 95% CI, 0.047-0.295; *P* = .007) and lower EQ-5D-3L summary index scores (β, −0.061; 95% CI, −0.105 to −0.017; *P* = .007) but not with higher SSM physical function scores (β, 0.068; 95% CI, −0.036 to 0.172; *P* = .20). The type of index operation was not significantly associated with the corresponding outcomes.

**Conclusions and Relevance:**

This cohort study showed no significant association between the type of index operation for DLSS—decompression alone or fusion—and the need for revision surgery or the outcomes of pain, disability, and quality of life among patients after 3 years. Number of revision operations was associated with more pain and worse quality of life.

## Key Points

**Question:**

Among patients with degenerative lumbar spinal stenosis (DLSS), is there an association between the type of index operation—decompression alone or decompression with fusion—and the incidence of revision surgery?

**Findings:**

In this cohort study of 328 patients, no significant association was found between the type of index operation for DLSS and the need for revision surgery after 3 years.

**Meaning:**

In this study, the type of index operation for DLSS was not associated with the incidence of revision surgery after 3 years.

## Teaser

This cohort study examines the association of decompression alone or decompression with fusion with incidence of revision surgery among patients with degenerative lumbar spinal stenosis.

## Introduction

There has been a remarkable increase in decompression and fusion surgery for lumbar spine disorders during the past 3 decades; numbers have increased by up to 3 fold,^1,2,3,4,5^ with the largest increase in patients aged 65 years or older.^2,5^ For degenerative lumbar spinal stenosis (DLSS), the most frequent indication for spine surgery in older adults,^6^ studies reported inconsistent rates of decompression without fusion procedures. Whereas US data showed a slight decrease between 2002 and 2009,^6,7^ data from Australia showed an increase between 2003 and 2013.^8^ In contrast, all 3 studies reported up to a 15-fold increase in fusion procedures.

The increase in operation rates could lead to more patients needing revision surgery, which is in general an undesired event owing to intraoperative or postoperative complications, bone fusion failures, persistence of pain, or additional progressive degeneration such as adjacent segments disease. The incidence of revision operations for patients with DLSS has been shown to range between 2.1% and 23% after 1 to 10 years of follow-up depending on the index procedure and study design.^9,10,11,12,13,14,15,16,17,18,19^ Furthermore, outcomes such as pain, disability, or satisfaction may be less favorable in patients with DLSS who undergo revision surgery than in those who do not.^17^ In addition, the need for revision surgery adds to the financial and resource burden for health care practitioners.

Data about the incidence of revisions in patients with DLSS derived from large cohort studies and the association of revision surgery with patient-reported outcome measures (PROMs) are scarce. Therefore, the aim of this study was to assess the cumulative incidence of revision operations after 2 types of index operations (ie, decompression alone or decompression with fusion) in patients with DLSS using 3-year follow-up data from a prospective cohort study, the Lumbar Stenosis Outcome Study (LSOS).^20^

## Methods

### Study Design

In this cohort study, we analyzed data from the LSOS,^20^ a prospective cohort study carried out at 8 spine surgery and rheumatology units in Switzerland that service a region with approximately 2 000 000 inhabitants. Patients aged 50 years or older with a history of neurogenic claudication were recruited between December 2010 and December 2015 and were followed up for 3 years. Patients with a significant deformity (>15° lumbar scoliosis) or a stenosis caused by a tumor, fracture, or infection were excluded. More information about the LSOS can be found elsewhere.^20,21^ The LSOS was conducted in compliance with all international laws and regulations as well as applicable guidelines. Each participant provided written informed consent to participate in the LSOS study and for their data to be further analyzed. The independent ethics committee of the Canton Zurich approved the LSOS study. The present study was approved by the Cantonal Ethics Commission Zurich. We followed the Strengthening the Reporting of Observational Studies in Epidemiology (STROBE) reporting guideline.^22^

### Inclusion and Exclusion Criteria

We included all patients who underwent either decompression alone or decompression with fusion surgery for DLSS and had 3 years of follow-up. We excluded patients from our analysis who underwent lumbar spine surgery before or more than 6 months after study enrollment, had more than 3 spinal disk levels decompressed, had a computed tomography scan only, or received only nonsurgical treatments.

### Surgical Technique or Approach

All patients underwent either decompression alone or decompression with fusion (hereafter referred to as fusion). Decompression surgery consisted of a standard open bilateral decompression or unilateral laminotomy with bilateral decompression of the affected disk level(s). Fusion surgery consisted of additional implantation of pedicle screws with rods plus intersomatic fusion and cage(s) at the affected disk level(s).

### Radiological Classification

Each magnetic resonance imaging scan at baseline was evaluated by 2 radiologists who verified and described the severity of DLSS by applying the 5 following core parameters according to a consensus paper^23^: compromise of the central zone,^24^ relation between the cerebrospinal fluid and the cauda equina (Schizas classification) for classification of central stenosis,^25^ nerve root compression in the lateral recesses for classification of lateral stenosis,^26^ foraminal nerve root impingement,^27^ and compromise of the foraminal zone for classification of foraminal stenosis.^24^ The Meyerding classification was used for grading the severity of the spondylolisthesis.^28^

### Patient-Reported Outcome Measures

#### Spinal Stenosis Measure

The Spinal Stenosis Measure (SSM) is a self-administered and validated questionnaire that was created for patients with DLSS to evaluate the severity of symptoms and patient disability.^29^ It is broadly used in studies investigating patients with DLSS^30,31,32,33^ and is recommended by the North American Spine Society.^34^ The SSM consists of the following 3 subscales: the symptom severity subscale (score range, 1-5, with higher scores indicating more pain), the physical function subscale (score range, 1-4, with higher scores indicating more disability), and the satisfaction subscale (score range, 1-4, with 1 indicating most satisfied).^35^

#### EuroQol Health-Related Quality of Life

The EuroQol Group developed the EuroQol Health-Related Quality of Life 5-Dimension 3-Level questionnaire (EQ-5D-3L), a self-administered standardized instrument to measure health-related quality of life (health state) in 5 dimensions (mobility, self-care, usual activities, pain or discomfort, and anxiety and/or depression) with 3 levels of severity for each dimension.^36^ The resulting health state can be transformed into a single summary index value using a value set that depends on population norms. For the French population, this value ranges from −0.53 to 1.00, calculated using the French time tradeoff, with 0 representing a health state equivalent to being dead and 1 indicating full health. We used the French value set to calculate the summary index because there is no specific value set for Switzerland.^37^

### Definition of Revision and Early Revision

Revision was defined as a renewed operation on at least 1 spinal level on which surgery was previously performed (index level[s]) or on an adjacent segment more than 3 months after the index operation. This 3-month period allowed adequate clinical evaluation and sufficient assessment of the implants after a fusion surgery and the healing of the bony structures. Repeated interventions that were performed within the 3-month period after the index operation owing to wound healing problems, infection, hematoma, or cerebrospinal fluid fistula were classified as early revisions.

### Clinical Outcomes

The primary outcome of our study was the cumulative incidence of revision operations. Secondary outcomes were changes in SSM symptom severity (pain) and SSM physical function (disability) subscale scores and the EQ-5D-3L summary index score (quality of life) between baseline and 1, 2, and 3 years of follow-up.

### Statistical Analysis

Data were analyzed from October to November 2021. We summarized the patient characteristics at baseline with medians and IQRs for continuous and ordinal variables and counts and percentages for categorical variables. To test for differences between the groups of patients at baseline, we used χ^2^ and Mann-Whitney tests and reported *P* values.

We used Kaplan-Meier estimators to compute the cumulative incidence (defined as 1 minus the Kaplan-Meier estimator) of revisions (events) for patients undergoing decompression alone and patients undergoing a fusion procedure at 1-, 2-, and 3-year follow-up. Furthermore, unadjusted absolute risk differences were calculated. The 95% CIs were obtained using the pooled variances of the 2 cumulative incidences.

We obtained the adjusted risk difference and the corresponding 95th-percentile bootstrap CI (2000 iterations) at 1, 2, and 3 years using a multivariable Cox proportional hazards regression model adjusting for the following clinically relevant variables as confounders, measured at baseline: age, sex, body mass index (≥25; calculated as weight in kilograms divided by height in meters squared), current smoker, civil risk (living alone or living in a nursing or residential home while being single, divorced, or widowed), duration of symptoms (>6 months), Cumulative Illness Rating Scale score,^38^ depression (Hospital Anxiety and Depression Scale [HADS]^39^ depression subscale score ≥8 points), anxiety (HADS anxiety subscale score ≥8 points), degenerative spondylolisthesis (Meyerding grade ≥I), stenotic disk levels (>1), and number of decompressed disk levels. We present the results of the multivariable Cox proportional hazards regression as hazard ratios (HRs) and corresponding 95% CIs. Furthermore, to consider specific clinically important patient characteristics, we performed subgroup analyses stratified by the number of decompressed disk levels and including only patients with degenerative spondylolisthesis (Meyerding grade ≥I).

To assess the association of PROMs with the number of revisions and the type of index operation (decompression alone or fusion), for each PROM, we used a linear mixed-effects model with patient-level random intercepts. The models included both variables of interest (number of revisions at the corresponding follow-up time and type of index operation) and the aforementioned clinically relevant confounders. We present the results as coefficient estimates (β) and corresponding 95% CIs.

All analyses were conducted with R, version 4.1.1 (R Project for Statistical Computing).^40^ Significance was set at 2-sided *P* < .05.

**Figure 1.**
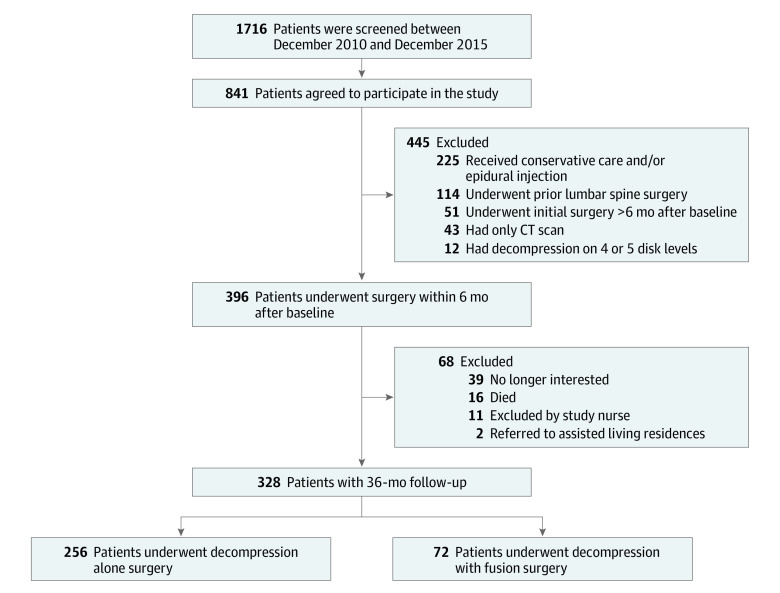
Study Flowchart CT indicates computed tomography.

We performed a sensitivity analysis to assess the robustness of our main outcome analysis, which was restricted to patients with complete 3-year follow-up. We therefore repeated the primary outcome analysis on an additional data set including patients without a complete follow-up of 3 years (ie, including patients who were no longer interested in participating in the study, had died, were excluded by a study nurse, or were referred to an assisted living residence) (Figure 1).

## Results

### Patient Characteristics

The study flowchart of the included patients is shown in Figure 1. For our analysis, 328 patients met the inclusion criteria (median [IQR] age, 73.0 years [66.0-78.0 years]; 165 [50.3%] male); of these, 256 (78.0%) underwent decompression alone and 72 (22.0%) underwent fusion.

Table 1 presents the patient characteristics at baseline. The median age was 75.0 years (IQR, 68.0-79.2 years) in the decompression alone group and 70.0 years (IQR, 64.0-74.0 years) in the fusion group (*P* < .001). Furthermore, more patients in the fusion group experienced symptoms for longer than 6 months (63 [87.5%] vs 192 [75.0%]; *P* = .04) and had an anxiety score of 8 points or more (23 [31.9%] vs 42 [16.4%]; *P* = .006) compared with patients in the decompression alone group.

**Table 1.** Baseline Characteristics of the Included Patients

Characteristic	Patients^a^			*P* value
	All (N = 328)	Decompression alone (n = 256)	Fusion (n = 72)	
Age, median (IQR), y	73.0 (66.0-78.0)	75.0 (68.0-79.2)	70.0 (64.0-74.0)	<.001
Sex				
Female	163 (49.7)	127 (49.6)	36 (50.0)	>.99
Male	165 (50.3)	129 (50.4)	36 (50.0)	
BMI				
Median (IQR)	26.8 (24.1-30.1)	26.7 (24.1-30.3)	27.4 (24.1-28.9)	.77
≥25	220 (67.1)	170 (66.4)	50 (69.4)	.73
Civil risk^b^	106 (32.3)	90 (35.2)	16 (22.2)	.054
Compulsory education^c^	78 (23.8)	65 (25.4)	13 (18.1)	.26
CIRS score, median (IQR)^d^	9.0 (7.0-12.0)	9.0 (7.0-12.0)	8.0 (6.0-11.2)	.08
Diabetes	32 (9.8)	29 (11.3)	3 (4.2)	.11
Current smoker	52 (15.9)	37 (14.5)	15 (20.8)	.26
Back pain	278 (84.8)	214 (83.6)	64 (88.9)	.36
Buttocks pain	259 (79.0)	202 (78.9)	57 (79.2)	>.99
Leg pain	297 (90.5)	235 (91.8)	62 (86.1)	.22
Strength or extent of complaints in the past 3 mo				
Getting better	20 (6.1)	18 (7.0)	2 (2.8)	.32
Staying about the same	52 (15.9)	43 (16.8)	9 (12.5)	
Getting worse	254 (77.4)	193 (75.4)	61 (84.7)	
Don’t know	2 (0.6)	2 (0.8)	0 (0.0)	
Duration of symptoms >6 mo	255 (77.7)	192 (75.0)	63 (87.5)	.04
HADS				
Depression score ≥8 points	54 (16.5)	43 (16.8)	11 (15.3)	.90
Anxiety score ≥8 points	65 (19.8)	42 (16.4)	23 (31.9)	.006
SSM				
Symptom severity score, median (IQR)^e^	3.1 (2.7-3.4)	3.1 (2.6-3.6)	3.1 (2.9-3.4)	.91
Physical function score, median (IQR)^f^	2.2 (1.8-2.8)	2.2 (1.8-2.8)	2.2 (1.8-2.6)	.51
EQ-5D-3L SI score, median (IQR)^g^	0.6 (0.2-0.7)	0.6 (0.2-0.7)	0.6 (0.3-0.7)	.58

Abbreviations: BMI, body mass index (calculated as weight in kilograms divided by height in meters squared); CIRS, Cumulative Illness Rating Scale; EQ-5D-3L SI, EuroQol Health-Related Quality of Life 5-Dimension 3-Level questionnaire summary index; HADS, Hospital Anxiety and Depression Scale; SSM, Spinal Stenosis Measure.

^a^ Data are presented as the number (percentage) of patients unless otherwise indicated.

^b^ Defined as living alone or as being single, divorced, or widowed and living in a nursing or residential home.

^c^ A required period of education imposed by the government of Switzerland.

^d^ Score ranges from 0 to 56; higher scores indicate greater severity.

^e^ Score ranges from 1 to 5; higher scores indicate more pain.

^f^ Score ranges from 1 to 4; higher scores indicate more disability.

^g^ Score ranges from −0.53 to 1.00; higher scores indicate better quality of life.

Most patients had at least 1 moderate grading on levels L4 to L5, followed by levels L3 to L4, and most had at least 3 stenotic levels (eTable 1 in Supplement 1). Furthermore, levels L4 to L5 were most affected by degenerative spondylolisthesis.

### Surgical Complications and Revisions

The most common perioperative surgical complications of the index operations were a dural tear (decompression alone, 18 [7.0%]; fusion, 2 [2.8%]) and postoperative wound infections (decompression alone, 4 [1.6%]; fusion, 1 [1.4%]) (Table 2). A total of 21 early revisions were performed in 19 patients; the median time difference between the index operation and the first early revision was 14.0 days (IQR, 5.0-24.0 days) in the decompression alone group and 6.0 days (IQR, 6.0-25.0 days) in the fusion group (Table 2).

**Table 2.** Index Operation, Perioperative and Postoperative Surgical Complications, Early Revisions, and Revisions

Variable	Decompression alone (n = 256)	Fusion (n = 72)	*P* value
Time between baseline and index operation, median (IQR) [range], d^a^	12.0 (3.0-33.0) [0-171]	23.0 (9.8-53.5) [1-180]	.003
Complications after index operation. No. (%)			
Intraoperative bleeding	4 (1.6)	0	.65
Intraoperative dural tear	18 (7.0)	2 (2.8)	.29
Postoperative wound infection	4 (1.6)	1 (1.4)	>.99
Postoperative osseous infection	1 (0.4)	0	>.99
Postoperative other^b^	21 (8.2)	6 (8.3)	.82
Early revisions, No. (%)^c^			
Total revisions	15 (5.9)	6 (8.3)	>.99
Patients with 1 revision	13 (5.1)	4 (5.6)	
Patients with 2 revisions	1 (0.4)	1 (1.4)	
Time between index operation and first early revision, median (IQR) [range], d	14.0 (5.0-24.0) [1-90]	6.0 (6.0-25.0) [2-49]	.71
Revisions, No. (%)			
Total revisions	37 (14.5)	11 (15.3)	.60
Patients with 1 revision	23 (9.0)	9 (12.5)	
Patients with 2 revisions	4 (1.6)	1 (1.4)	
Patients with 3 revisions	2 (0.8)	0	
Indication for revision, No./total No. (%)			
Restenosis	36/37 (97.3)	3/11 (27.3)	<.001
Infection	1/37 (2.7)	0	>.99
Epidural bleeding	0	0	NA
Other^d^	0	8/11 (72.7)	<.001
Type of revision, No./total No. (%)			
Decompression alone	5/37 (13.5)	3/11 (27.3)	.54
Fusion	32/37 (86.5)	8/11 (72.7)	
Complications after revisions, No./total No. (%)			
Intraoperative bleeding	0	0	NA
Intraoperative dural tear	2/37 (5.4)	1/11 (9.1)	>.99
Postoperative wound infection	1/37 (2.7)	0	>.99
Postoperative osseous infection	0	0	NA
Postoperative other^b^	3/37 (8.1)	1/11 (9.1)	>.99
Time between index operation and first revision, median (IQR) [range], d^e^	402.0 (202.0-565.0) [91-931]	549.5 (419.5-754.8) [97-869]	.13
Time between baseline and follow-up, median (IQR), d			
1 y	365.0 (365.0-368.0)	365.0 (365.0-366.0)	.33
2 y	731.0 (730.0-732.0)	731.0 (730.0-731.0)	.85
3 y	1096.0 (1096.0-1097.0)	1096.0 (1096.0-1097.0)	.34

Abbreviation: NA, not applicable.

^a^ Baseline was the entry date into study.

^b^ For example, urosepsis, hemorrhage, or wound healing deficit.

^c^ Repeated intervention within 3 months after the index operation owing to wound healing problems, infection, hematoma, or cerebrospinal fluid fistula.

^d^ Screw loosening, screw misalignment, or pedicle fracture.

^e^ Includes only patients who underwent revision operations.

A total of 48 revisions were performed in 39 patients; of these, 7 patients (18.0%) had 2 or more revisions. The median time difference between the index operation and the first revision was 402.0 days (IQR, 202.0-565.0 days) in the decompression alone group and 549.5 days (IQR, 419.5-754.8 days) in the fusion group. The most common indication for revision in the decompression alone group was restenosis (36 operations [97.3%]), and the most common indication in the fusion group was screw loosening (6 operations [54.5%]). In both groups, most revision surgeries included fusion procedures (decompression alone, 32 [86.5%]; fusion, 8 [72.7%]), with the postoperative complications being similar to those after the index operation (Table 2).

### Primary Outcome Measure

**Figure 2.**
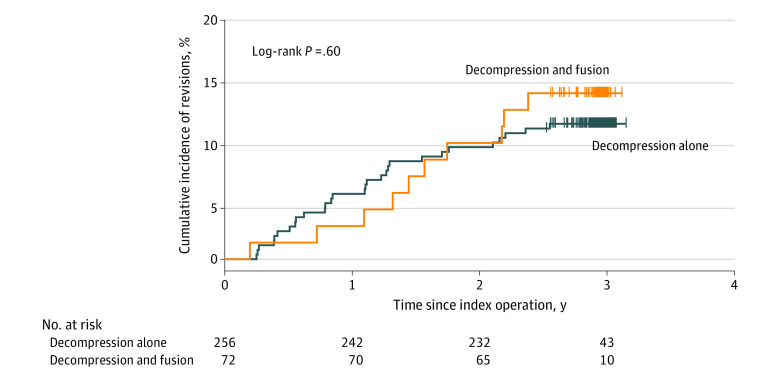
Kaplan-Meier Curve of the Cumulative Incidence of Revisions The date of the initial surgery was considered the index date. Revision (event) was defined as a renewed operation on at least 1 spinal disk level on which surgery was previously performed (index level[s]) or on an adjacent segment more than 3 months after the index operation.

The cumulative incidence of revisions after 3 years was 11.3% (95% CI, 7.4%-15.1%) for the decompression alone group and 13.9% (95% CI, 5.5%-21.5%) for the fusion group (log-rank *P* = .60) (Figure 2). There was no significant difference in the need for revision between the 2 groups over time. The unadjusted absolute risk difference was 2.6% (95% CI, −6.3% to 11.4%), the adjusted absolute risk difference was 3.9% (95% CI, −5.2% to 17.0%), and the adjusted HR was 1.40 (95% CI, 0.63-3.13; *P* = .41). The corresponding numbers for 1- and 2-year follow-up are presented in Table 3.

**Table 3.** Cumulative Incidence of Revisions

Time after index operation	Decompression alone		Fusion		Risk difference, % (95% CI)		*P* value^c^
	Cumulative incidence, % (95% CI)^a^	Revisions, No./total No. of patients at risk^b^	Cumulative incidence, % (95% CI)^a^	Revisions, No./total No. of patients at risk^b^	Absolute^a^	Adjusted absolute^c^^,^^d^	
At index operation	NA	0/256	NA	0/72	NA	NA	NA
1 y	5.5 (2.6 to 8.2)	14/242	2.8 (0.0 to 6.5)	2/70	−2.7 (−7.4 to 2.0)	1.7 (−2.3 to 8.5)	.51
2 y	9.4 (5.7 to 12.9)	24/232	9.7 (2.6 to 16.3)	7/65	0.3 (−7.4 to 8.1)	3.2 (−4.3 to 14.7)	.50
3 y	11.3 (7.4 to 15.1)	29/43	13.9 (5.5 to 21.5)	10/10	2.6 (−6.3 to 11.4)	3.9 (−5.2 to 17.0)	.49

Abbreviation: NA, not applicable.

^a^ Unadjusted estimates.

^b^ Cumulative numbers of revisions among patients who underwent the procedure.

^c^ From the fully adjusted multivariate Cox proportional hazards regression model using the variables age, sex, body mass index (≥25; calculated as weight in kilograms divided by height in meters squared), current smoker, civil risk (living alone or living in a nursing or residential home while being single, divorced, or widowed), duration of symptoms (>6 months), Cumulative Illness Rating Scale score, depression (Hospital Anxiety and Depression Scale [HADS] depression subscale score ≥8 points), anxiety (HADS anxiety subscale score ≥8 points), degenerative spondylolisthesis (Meyerding grade≥I), stenotic levels (>1 stenotic level), and number of decompressed levels.

^d^ The 95% CIs were obtained by the adjusted bootstrap percentile method from 2000 bootstrap iterations.

In the subgroup analyses, we observed no significant difference in experiencing revision between the 2 groups over time when stratified by number of decompressed levels at the index operation (eFigure in Supplement 1) or when only patients with degenerative spondylolisthesis were included (eTable 2 in Supplement 1).

eTable 3 in Supplement 1 shows the results of the multivariate Cox proportional hazards regression model. Duration of symptoms of more than 6 months was associated with lower risk of revision (HR, 0.45; 95% CI, 0.22-0.91; *P* = .03), and depression was associated with higher risk of revision (HR, 2.32; 95% CI, 1.02-5.29; *P* = .046).

### Secondary Outcome Measures

To assess the association of number of revisions and type of index operation with SSM symptom severity and physical function subscale scores and the EQ-5D-3L summary index score, mixed-effects models were fitted with patient-level random intercepts. On average, patients reported significantly improved clinical outcomes over time (eTable 4 in Supplement 1). The number of revisions was associated with higher SSM symptom severity scores (β, 0.171; 95% CI, 0.047-0.295; *P* = .007) and lower EQ-5D-3L summary index scores (β, −0.061; 95% CI, −0.105 to −0.017; *P* = .007), meaning that undergoing more operations was associated with worse outcomes. However, there was no evidence of an association of number of revisions with higher SSM physical function scores (β, 0.068; 95% CI, −0.036 to 0.172; *P* = .20). The type of index operation (fusion or decompression alone), number of decompressed levels, and presence of degenerative spondylolisthesis were not significantly associated with the corresponding outcomes. Female sex, body mass index, Cumulative Illness Rating Scale score, and depression were negatively associated with the secondary outcomes. Anxiety was also negatively associated with SSM symptom severity scores, age with SSM physical function scores, and civil risk with EQ-5D-3L summary index scores.

### Sensitivity Analysis

The sensitivity analysis included 396 patients. Of these, 309 (78.0%) underwent decompression alone and 87 (22.0%) underwent a fusion procedure (eTable 5 in Supplement 1). The results were consistent with those of the main analysis (Table 3 and eTables 6 and 7 in Supplement 1).

## Discussion

In this cohort study, we found no evidence of a significant difference in the need for revision between decompression alone and fusion surgery after 3 years in patients with DLSS. Undergoing revision was associated with experiencing more pain and worse quality of life, whereas no evidence of an association between undergoing revision and more disability was found. The type of index operation (ie, decompression with or without fusion) was not significantly associated with the corresponding outcomes.

In the past 5 years, 3 randomized clinical trials (1 from the US^13^ and 2 from Europe^10,12^) investigating patients with symptomatic lumbar spinal stenosis undergoing decompression alone or fusion procedures were published. The 3 trials found considerably different incidences of revision. Ghogawala et al^13^ reported a 34% incidence of revision in the decompression alone group and a 14% incidence of revision in the fusion group at 4-year follow-up; Försth et al^12^ reported incidences of 21% and 22% in the decompression alone and fusion groups, respectively, after a mean follow-up of 6.5 years; and Austevoll et al^10^ reported incidences of 12.5% and 9.1% in the decompression alone and fusion groups, respectively, at 2-year follow-up. With increasing length of study, the incidence of revision increased, as we also found in our own study (Table 3); this may in part explain the difference in incidences of revision between the studies. The higher incidence of revision in the decompression alone group in the US trial (34%)^13^ was possibly owed to the decompression procedure performed (ie, complete laminectomy). In comparison, in the trial by Austevoll et al^10^ and in our study, patients underwent only laminotomies. The difference in the incidences of revision in the fusion groups between all studies might in part be explained by the patient sample. Austevoll et al^10^ and Ghogawala et al^13^ only included patients with spondylolisthesis, whereas Försth et al^12^ and our study included patients with and without spondylolisthesis.

Our study also demonstrated that the incidence of revision was not significantly associated with the type of index surgery. Similar results were reported by Austevoll et al^10^ and by 3 studies based on claims data.^11,18,41^ The different study designs of randomized clinical trials and observational studies gain complementary evidence regarding how surgical treatments work in real-life clinical situations.

Results of the Spine Patient Outcomes Research Trial (SPORT) showed that patients who had undergone revision experienced more disability and pain during up to 8 years of follow-up.^17^ Although we did not find any increase in disability, that finding was consistent with our results. In addition, our study found significant negative associations between depression and all our PROMs and risk of revision. In a retrospective review, Miller et al^42^ similarly demonstrated an association between depression and a significant reduction in quality of life after surgery. In an analogous prospective observational 10-year follow-up study, Tuomainen et al^43^ reported an association between depression and an increased likelihood of pain and disability in a sample of patients with DLSS and depression.

The association between a lower incidence of revision and the duration of symptoms before the index surgery suggests that more sustainable outcomes may be associated with surgical decisions being made later. In other words, earlier and perhaps more hasty decisions may have been made without the necessary deliberation.

Decisions about the type of index operation and whether to perform revision are likely related to the experience of the individual surgeon in terms of his or her training and surgical approach. This factor might explain some of the variation in the procedures performed among patients with almost identical conditions by different surgeons.

Furthermore, the variation in incidence of revision may be associated with other variables (eg, trust and communication) that emerge in the patient-practitioner relationship during the discussion of treatment. In addition, the incidence of revision in the first year may be particularly associated with the time needed for bone healing as well as the general preference among surgeons to wait for at least 1 year after surgery before declaring failure of a fusion.

Future research avenues include gathering more data to gain a clearer picture of incidence of revision to facilitate decision-making among patients with DLSS and their surgeons. Establishing what constitutes an unfavorable outcome in a revised surgical decompression with or without fusion and linking it to patient-level factors would aid in the development of guidelines to evaluate patients before surgery, stratify risks, predict outcomes, and make shared decisions.

### Limitations

This study has limitations. The main limitation was the heterogeneous patient sample with differences in some variables, such as age, duration of symptoms, anxiety, or number of stenotic spinal levels at baseline. To mitigate these limitations, we used multivariable linear mixed-effects models and adjusted for the corresponding confounders. Nonetheless, in observational studies, unobserved baseline confounders between the groups can bias the results. Second, considering the wide range of the 95% CIs for the adjusted absolute risk difference and HRs, a trend toward a more frequent risk of revisions in fused spines may have guided the clinicians toward a restrained indication for fusion in cases of borderline situations in which the advantages and disadvantages of both decompression alone and decompression and fusion were opposed. Third, we excluded patients without 3-year follow-up; however, the results of the sensitivity analysis including patients without complete 3-year follow-up were consistent with those of the main analysis. Fourth, as in all observational studies, we cannot rule out the risk of selection bias (eg, the choice of approach might have been influenced by patient characteristics such as age or number of stenotic levels). Fifth, the fusion group was smaller than the decompression alone group.

## Conclusions

Our study showed that there was no significant association between the type of index operation—decompression alone or fusion—and the need for revision surgery or the outcomes of pain, disability, and quality of life after 3 years in patients with DLSS. Number of revision operations was associated with more pain and worse quality of life.

## Notes

Supplement 1. **eTable 1.** Radiologic Evaluations of the MRIs **eTable 2.** Cumulative Incidence of Revisions and 95% Confidence Intervals, Including Only Patients With Degenerative Spondylolisthesis **eTable 3.** Multivariable Cox Proportional Hazards Regression Model **eTable 4.** Mixed-effects Models for the Outcomes SSM Symptoms, SSM Function, and EQ-5D-3L SI **eTable 5.** Sensitivity Analysis: Baseline Characteristics of the Included Patients **eTable 6.** Sensitivity Analysis: Cumulative Incidence of Revisions and 95% Confidence Intervals **eTable 7.** Sensitivity Analysis: Multivariate Cox Proportional Hazards Regression Model **eFigure.** Cumulative Incidence of Revisions (Kaplan-Meier), Stratified into Number of Decompressed Levels

Supplement 2.
Lumbar Stenosis Outcome Study Group
