# Supplementary material for: Incidence of Revision Surgery After Decompression With vs Without Fusion Among Patients With Degenerative Lumbar Spinal Stenosis
Source: JAMA Netw Open. 2022 Jul 26;5(7):e2223803. doi: 10.1001/jamanetworkopen.2022.23803 (PMC9327572; doi:10.1001/jamanetworkopen.2022.23803)
Supplement: Supplement 1. — eTable 1. Radiologic Evaluations of the MRIs eTable 2. Cumulative Incidence of Revisions and 95% Confidence Intervals, Including Only Patients With Degenerative Spondylolisthesis eTable 3. Multivariable Cox Proportional Hazards Regression Model eTable 4. Mixed-effects Models for the Outcomes SSM Symptoms, SSM Function, and EQ-5D-3L SI eTable 5. Sensitivity Analysis: Baseline Characteristics of the Included Patients eTable 6. Sensitivity Analysis: Cumulative Incidence of Revisions and 95% Confidence Intervals eTable 7. Sensitivity Analysis: Multivariate Cox Proportional Hazards Regression Model eFigure. Cumulative Incidence of Revisions (Kaplan-Meier), Stratified into Number of Decompressed Levels [file jamanetwopen-e2223803-s001.pdf]

Supplementary Online Content

Ulrich NH, Burgstaller JM, Valeri F, et al; Lumbar Stenosis Outcome Study Group. Incidence of revision surgery after decompression with vs without fusion among patients with degenerative lumbar spinal stenosis. *JAMA Netw Open*. 2022;5(7):e2223803. doi:10.1001/jamanetworkopen.2022.23803

eTable 1. Radiologic Evaluations of the MRIs

eTable 2. Cumulative Incidence of Revisions and 95% Confidence Intervals, Including Only Patients With Degenerative Spondylolisthesis

eTable 3. Multivariable Cox Proportional Hazards Regression Model

eTable 4. Mixed-effects Models for the Outcomes SSM Symptoms, SSM Function, and EQ-5D-3L SI

eTable 5. Sensitivity Analysis: Baseline Characteristics of the Included Patients

eTable 6. Sensitivity Analysis: Cumulative Incidence of Revisions and 95% Confidence Intervals

eTable 7. Sensitivity Analysis: Multivariate Cox Proportional Hazards Regression Model

eFigure. Cumulative Incidence of Revisions (Kaplan-Meier), Stratified into Number of Decompressed Levels

This supplementary material has been provided by the authors to give readers additional information about their work.

© 2022 Ulrich NH et al. *JAMA Network Open*.

eTable 1: Radiologic evaluations of the MRIs

Variable	Decompression alone	Fusion	p-value
	No (%)	No (%)	
No. of patients	256	72	
Stenosis levels[a]			
L1/L2	58 (22.7)	8 (11.1)	**0.046**
L2/L3	130 (50.8)	26 (36.1)	**0.039**
L3/L4	201 (78.5)	39 (54.2)	**<0.001**
L4/L5	228 (89.1)	63 (87.5)	0.873
L5/S1	109 (42.6)	28 (38.9)	0.670
Stenotic levels[a]			**0.010**
0	2 (0.8)	1 (1.4)	
1	32 (12.5)	20 (27.8)	
2	68 (26.6)	20 (27.8)	
≥3	154 (60.2)	31 (43.1)	
Spondylolisthesis[b], n levels (%)			
L1/L2	10 (3.9)	3 (4.2)	1
L2/L3	19 (7.4)	5 (6.9)	1
L3/L4	50 (19.5)	15 (20.8)	0.938
L4/L5	95 (37.1)	45 (62.5)	**<0.001**
L5/S1	46 (18.0)	16 (22.2)	0.520

[a] at least one moderate grading in one of the following core parameters: compromise of the central zone, relation between fluid and cauda equina (Schizas classification), and nerve root compression in the lateral recesses

[b] Meyerding listhesis grade ≥1

© 2022 Ulrich NH et al. *JAMA Network Open*.

eTable 2: Cumulative incidence of revisions and 95% confidence intervals, including only patients with degenerative spondylolisthesis

	**Decompression alone**		**Fusion**				
Time after index operation	Cumulative incidence, % (95% CI)[a]	No. at risk / of revisions[b]	Cumulative incidence, % (95% CI)[a]	No. at risk / of revisions[b]	Absolute risk difference, % (95% CI)[a]	Adj. absolute risk difference, % (95% CI)[c,d]	P value [c]
at index operation	n.a. (n.a.)	146 / 0	n.a. (n.a.)	57 / 0	n.a. (n.a.)	n.a. (n.a.)	n.a.
1 year	6.2 (2.2-10.0)	137 / 9	1.8 (0.0-5.1)	56 / 1	-4.4 (-9.6-0.8)	1.1 (-5.2-10.0)	0.763
2 years	9.6 (4.7-14.2)	132 / 14	10.5 (2.2-18.1)	51 / 6	0.9 (-8.4-10.2)	2.1 (-8.9-16.8)	0.750
3 years	11.0 (5.7-15.9)	26 / 16	10.5 (2.2-18.1)	8 / 6	-0.5 (-9.9-9.0)	2.3 (-9.7-18.0)	0.747

Abbreviations: adj., adjusted; CI, confidence interval

[a] Unadjusted estimates.

[b] Cumulative numbers of revisions.

[c] From fully adjusted multivariate Cox proportional hazards regression model (using the variables age, sex, body mass index (BMI) (≥25 kg/m2), current smoker, civil risk (living alone, or living in a nursing/residential home while being single, divorced, or widowed), duration of symptoms (>6 months), cumulative illness rating scale (CIRS), depression (Hospital Anxiety and Depression Scale (HADS) depression subscale ≥8 points), anxiety (HADS anxiety subscale ≥8 points), degenerative spondylolisthesis (Meyerding grade ≥I), stenotic levels (>1 stenotic level), and number of decompressed levels).

[d] The 95% CIs were obtained by the adjusted bootstrap percentile method from 2000 bootstrap iterations.

© 2022 Ulrich NH et al. *JAMA Network Open*.

eTable 3: Multivariable Cox proportional hazards regression model

**Covariate**	**Hazard ratio (95% CI)**	**P value**
Fusion (type of index operation)	1.40 (0.63-3.13)	0.409
Age (centered)	0.98 (0.94-1.02)	0.377
Female	0.82 (0.39-1.70)	0.593
BMI (≥25kg/m2)	1.18 (0.56-2.53)	0.662
Civil risk[a]	0.90 (0.41-1.98)	0.788
Current smoker	0.65 (0.24-1.76)	0.395
Duration of symptoms (>6 months)	0.45 (0.22-0.91)	**0.026**
CIRS (centered)	1.04 (0.95-1.14)	0.418
Depression	2.32 (1.02-5.29)	**0.046**
Anxiety	1.62 (0.73-3.61)	0.236
Degenerative spondylolisthesis (Meyerding Grade ≥I)	0.77 (0.39-1.51)	0.446
Number of stenotic levels (>1 level)	0.92 (0.34-2.49)	0.863
2 Decompressed levels	1.48 (0.65-3.37)	0.355
3 Decompressed levels	1.82 (0.71-4.67)	0.214

Overall tests: likelihood ratio test p=0.09

Abbreviations: BMI, body mass index; CI, confidence interval; CIRS, Cumulative Illness Rating Scale
[a] Living alone, or single/divorced/widowed and living in a nursing/residential home

© 2022 Ulrich NH et al. *JAMA Network Open*.

eTable 4: Mixed-effects models for the outcomes SSM symptoms, SSM function, and EQ-5D-3L SI

A) SSM symptoms

	**Estimate**	**Lower 95% CI**	**Upper 95% CI**	**p-value**
(Intercept)	2.731	2.497	2.965	**0.000**
Cumulative number of total operations	0.171	0.047	0.295	**0.007**
Fusion (type of index operation)	-0.1	-0.258	0.057	0.214
Age (centered)	0.007	-0.002	0.015	0.112
Female	0.227	0.089	0.364	**0.001**
BMI (≥25kg/m^2)	0.185	0.054	0.315	**0.006**
Civil risk	-0.023	-0.169	0.124	0.760
Duration of symptoms (>6 months)	0.129	-0.02	0.277	0.090
CIRS (centered)	0.031	0.015	0.048	**0.000**
Current smoker	0.08	-0.092	0.253	0.364
Depression (≥8 points)	0.367	0.184	0.55	**0.000**
Anxiety (≥8 points)	0.189	0.018	0.36	**0.031**
Degenerative spondylolisthesis (Meyerding Grade ≥I)	-0.067	-0.198	0.063	0.312
Number of stenotic levels (>1 level)	0.03	-0.149	0.21	0.742
2 decompressed levels	-0.026	-0.169	0.116	0.716
3 decompressed levels	0.04	-0.146	0.225	0.677
Follow-up 12 months	-1.216	-1.367	-1.064	**0.000**
Follow-up 24 months	-1.203	-1.361	-1.044	**0.000**
Follow-up 36 months	-1.21	-1.372	-1.048	**0.000**

© 2022 Ulrich NH et al. *JAMA Network Open*.

B) SSM function

	Estimate	Lower 95% CI	Upper 95% CI	p-value
(Intercept)	2.037	1.848	2.226	**0.000**
Cumulative number of total operations	0.068	-0.036	0.172	0.198
Fusion (type of index operation)	-0.125	-0.252	0.002	0.055
Age (centered)	0.007	0.001	0.014	**0.032**
Female	0.213	0.102	0.324	**0.000**
BMI (≥25kg/m^2)	0.192	0.088	0.297	**0.000**
Civil risk	-0.03	-0.148	0.088	0.624
Duration of symptoms (>6 months)	0.002	-0.117	0.122	0.970
CIRS	0.016	0.003	0.03	**0.015**
Current smoker	0.055	-0.084	0.194	0.440
Depression (≥8 points)	0.399	0.251	0.546	**0.000**
Anxiety (≥8 points)	0.025	-0.112	0.163	0.718
Degenerative spondylolisthesis (Meyerding Grade ≥I)	-0.061	-0.166	0.044	0.258
Number of stenotic levels (>1 level)	-0.046	-0.19	0.099	0.536
2 decompressed levels	0.007	-0.108	0.122	0.908
3 decompressed levels	0.012	-0.138	0.161	0.878
Follow-up 12 months	-0.816	-0.944	-0.689	**0.000**
Follow-up 24 months	-0.798	-0.931	-0.665	**0.000**
Follow-up 36 months	-0.774	-0.91	-0.638	**0.000**

© 2022 Ulrich NH et al. *JAMA Network Open*.

C) EQ-5D-3L SI

	Estimate	Lower 95% CI	Upper 95% CI	p-value
(Intercept)	0.628	0.554	0.703	**0.000**
Cumulative number of total operations	-0.061	-0.105	-0.017	**0.007**
Fusion (type of index operation)	0.029	-0.021	0.079	0.251
Age (centered)	-0.001	-0.004	0.001	0.321
Female	-0.098	-0.142	-0.055	**0.000**
BMI (≥25kg/m^2)	-0.062	-0.103	-0.02	**0.004**
Civil risk	0.047	0.001	0.094	**0.046**
Duration of symptoms (>6 months)	-0.02	-0.067	0.027	0.396
CIRS	-0.01	-0.015	-0.005	**0.000**
Current smoker	-0.023	-0.078	0.032	0.413
Depression (≥8 points)	-0.172	-0.23	-0.114	**0.000**
Anxiety (≥8 points)	-0.031	-0.085	0.023	0.258
Degenerative spondylolisthesis (Meyerding Grade ≥I)	0.036	-0.006	0.077	0.093
Number of stenotic levels (>1 level)	-0.012	-0.069	0.045	0.677
2 decompressed levels	-0.006	-0.051	0.039	0.805
3 decompressed levels	-0.037	-0.096	0.021	0.213
Follow-up 12 months	0.334	0.279	0.389	**0.000**
Follow-up 24 months	0.329	0.272	0.386	**0.000**
Follow-up 36 months	0.316	0.258	0.375	**0.000**

© 2022 Ulrich NH et al. *JAMA Network Open*.

eTable 5: Sensitivity analysis: baseline characteristics of the included patients

Variable	All	Decompression alone	Fusion	p-value
	No (%)	No (%)	No (%)	
No. of patients	396	309	87	
Age, years, median (IQR)	74.0 [67.0, 79.0]	75.0 [68.0, 80.0]	70.0 [64.0, 74.0]	**<0.001**
Female	200 (50.5)	157 (50.8)	43 (49.4)	0.915
BMI, kg/m^2, median (IQR)	26.7 [24.0, 30.0]	26.4 [24.0, 30.2]	27.4 [24.0, 28.7]	0.605
BMI ≥25, kg/m^2	263 (66.4)	202 (65.4)	61 (70.1)	0.485
Civil Risk[a]	134 (33.8)	112 (36.2)	22 (25.3)	0.075
Compulsory education	102 (25.8)	84 (27.2)	18 (20.7)	0.278
CIRS, median (IQR)	9.0 [7.0, 12.0]	9.0 [7.0, 12.0]	8.0 [6.0, 11.5]	**0.039**
Diabetes mellitus	41 (10.4)	37 (12.0)	4 (4.6)	0.073
Current smoker	58 (14.6)	40 (12.9)	18 (20.7)	0.102
Back pain	340 (85.9)	263 (85.1)	77 (88.5)	0.530
Buttocks pain	308 (77.8)	243 (78.6)	65 (74.7)	0.527
Leg pain	359 (90.7)	284 (91.9)	75 (86.2)	0.160
Problem getting better or worse in the last 3 months				0.218
Getting better	23 (5.8)	21 (6.8)	2 (2.3)	
Staying about the same	63 (15.9)	52 (16.8)	11 (12.6)	
Getting worse	308 (77.8)	234 (75.7)	74 (85.1)	
Don't know	2 (0.5)	2 (0.6)	0 (0.0)	
Duration of symptoms >6 months	299 (75.5)	226 (73.1)	73 (83.9)	0.055
HADS depression (≥8 points)	70 (17.7)	59 (19.1)	11 (12.6)	0.217
HADS anxiety (≥8 points)	80 (20.2)	55 (17.8)	25 (28.7)	**0.036**
SSM symptoms, median (IQR)[b]	3.1 [2.7, 3.6]	3.1 [2.7, 3.6]	3.1 [2.8, 3.4]	0.893
SSM function, median (IQR)[c]	2.2 [1.8, 2.8]	2.2 [1.8, 2.8]	2.2 [1.8, 2.6]	0.141
EQ-5D-3L SI, median (IQR)[d]	0.6 [0.2, 0.7]	0.6 [0.2, 0.7]	0.6 [0.4, 0.7]	0.979

Abbreviations: BMI, body mass index; CIRS, Cumulative Illness Rating Scale; IQR, interquartile range
[a] Living alone, or single/divorced/widowed and living in a nursing/residential home.
[b] score range 1-4 (best-worst): higher scores indicate more pain.
[c] score range 1-5 (best-worst); higher scores indicate more disability.
[d] score range -0.53-1.00 (worst-best); higher scores indicate better quality of life.

© 2022 Ulrich NH et al. *JAMA Network Open*.

eTable 6: Sensitivity analysis: cumulative incidence of revisions and 95% confidence intervals

	**Decompression alone**		**Fusion**				
Time after index operation	Cumulative incidence, % (95% CI)[a]	No. at risk / of revisions[b]	Cumulative incidence, % (95% CI)[a]	No. at risk / of revisions[b]	Absolute risk difference, % (95% CI)[a]	Adj. absolute risk difference, % (95% CI)[c,d]	P value[c]
at index operation	n.a. (n.a.)	309 / 0	n.a. (n.a.)	87 / 0	n.a. (n.a.)	n.a. (n.a.)	n.a.
1 year	5.1 (2.5-7.5)	269 / 15	3.6 (0.0-7.5)	79 / 3	-1.5 (-6.1-3.2)	2.5 (-1.4-9.4)	0.335
2 years	8.7 (5.4-11.8)	243 / 25	9.7 (3.1-15.9)	70 / 8	1.0 (-6.0-8.2)	4.5 (-2.6-15.4)	0.316
3 years	10.9 (7.2-14.5)	46 / 31	13.7 (5.8-20.9)	10 / 11	2.8 (-5.4-11.1)	5.7 (-3.2-18.5)	0.305

Abbreviations: adj., adjusted; CI, confidence interval
In the sensitivity analysis also the patients without a complete follow-up of three years were included in the analysis (see Figure 1), i.e., “no longer interested” (n = 39), “died” (n = 16), “excluded by study nurse” (n = 11), “referred to assisted living residences” (n = 2).
[a] Unadjusted estimates.
[b] Cumulative numbers of revisions.
[c] From fully adjusted multivariate Cox proportional hazards regression model (using the variables age, sex, body mass index (BMI) (≥25 kg/m2), current smoker, civil risk (living alone, or living in a nursing/residential home while being single, divorced, or widowed), duration of symptoms (>6 months), cumulative illness rating scale (CIRS), depression (Hospital Anxiety and Depression Scale (HADS) depression subscale ≥8 points), anxiety (HADS anxiety subscale ≥8 points), degenerative spondylolisthesis (Meyerding grade ≥I), stenotic levels (>1 stenotic level), and number of decompressed levels).
[d] The 95% CIs were obtained by the adjusted bootstrap percentile method from 2000 bootstrap iterations.

© 2022 Ulrich NH et al. *JAMA Network Open*.

eTable 7: Sensitivity analysis: multivariable Cox proportional hazards regression model

Covariate	Hazard ratio (95% CI)	p-value
Fusion (type of index operation)	1.62 (0.76-3.46)	0.213
Age (centered)	0.99 (0.95-1.03)	0.592
Female	0.83 (0.41-1.67)	0.593
BMI (≥25kg/m2)	1.04 (0.51-2.12)	0.915
Civil risk[a]	0.93 (0.44-1.95)	0.852
Current smoker	0.61 (0.22-1.67)	0.336
Duration of symptoms (>6 months)	0.41 (0.21-0.80)	**0.008**
CIRS (centered)	1.05 (0.97-1.14)	0.257
Depression	2.42 (1.09-5.37)	**0.030**
Anxiety	1.40 (0.64-3.06)	0.405
Degenerative spondylolisthesis (Meyerding Grade ≥I)	0.79 (0.41-1.52)	0.486
Number of stenotic levels (>1 level)	0.93 (0.34-2.49)	0.878
2 Decompressed levels	1.47 (0.65-3.29)	0.354
3 Decompressed levels	2.22 (0.91-5.44)	0.081

Overall tests: likelihood ratio test p=0.09
Abbreviations: BMI, body mass index; CI, confidence interval; CIRS, Cumulative Illness Rating Scale
In the sensitivity analysis also the following primarily excluded patients were included (see Figure 1): “no longer interested” (n = 39), “died” (n = 16), “excluded by study nurse” (n = 11), “referred to assisted living residences” (n = 2).
[a] Living alone, or single/divorced/widowed and living in a nursing/residential home

© 2022 Ulrich NH et al. *JAMA Network Open*.

eFigure: Cumulative incidence of revisions (Kaplan-Meier), stratified into number of decompressed levels

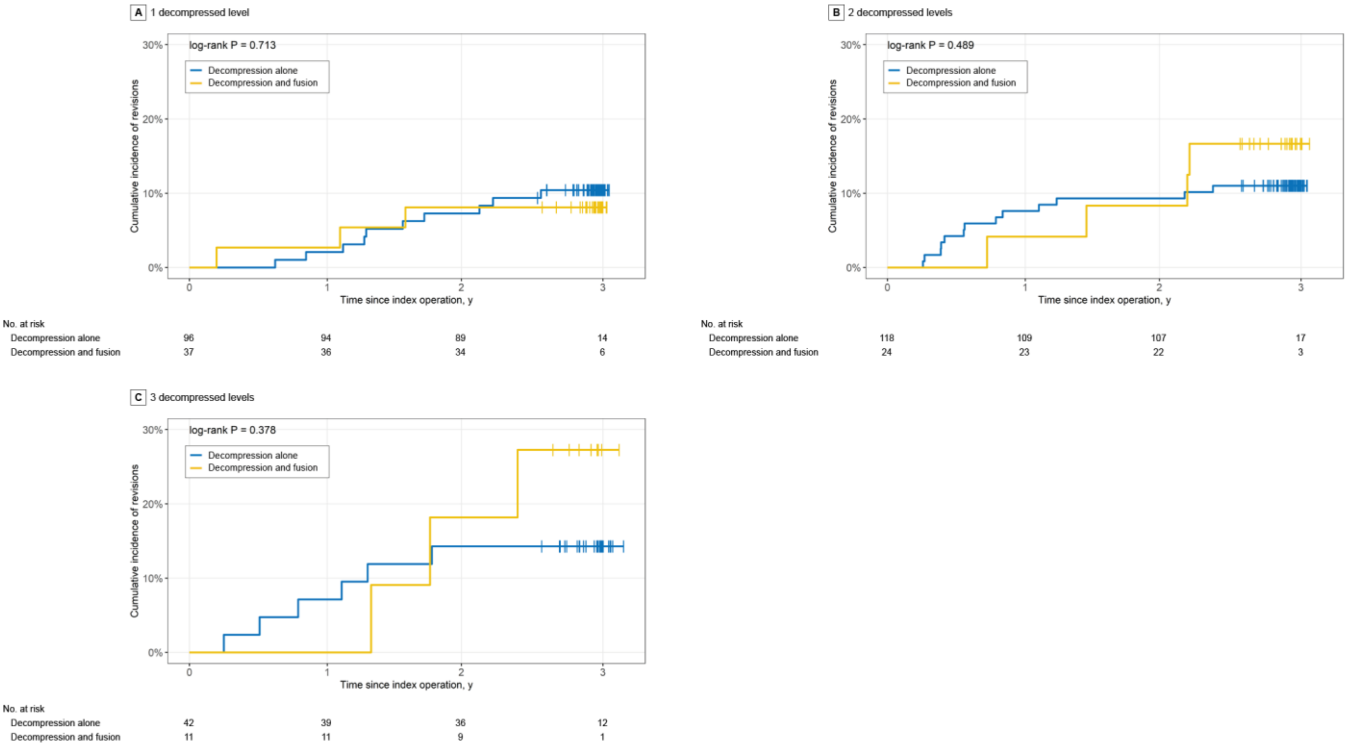

The date of the initial surgery was considered as the index date. Revision (event) is defined as a renewed operation on at least one level that was operated before (index level(s)) or on an adjacent segment after three months of the index operation.

Patients were stratified into number of decompressed levels, e.g., patients in the decompression alone group that underwent surgery only on 1 level were compared to patients in the fusion group that underwent surgery only on 1 level.

© 2022 Ulrich NH et al. *JAMA Network Open*.
