# Supplementary material for: Incidence of Revision Surgery After Decompression With vs Without Fusion Among Patients With Degenerative Lumbar Spinal Stenosis
Source: JAMA Netw Open. 2022 Jul 26;5(7):e2223803. doi: 10.1001/jamanetworkopen.2022.23803 (PMC9327572; doi:10.1001/jamanetworkopen.2022.23803)
Supplement: Supplement 2. — Lumbar Stenosis Outcome Study Group [file jamanetwopen-e2223803-s002.pdf]

Supplemental Online Content: Nonauthor Collaborators

*First name, last name, and suffix (if applicable) are required and will appear in PubMed.

*Group Name(s): Lumbar Stenosis Outcome Study Group							
***First Name and Middle Initial(s)**	***Last Name**	***Suffix (eg, Jr, III)**	Academic Degrees	Institution	Location (city, state/province, country)	Role or Contribution, eg, chair, principal investigator	Group (if more than 1 Group listed in the byline) and/or Subgroup (eg, Steering Committee)
Florian	Brunner		Prof	University Hospital Balgrist	Zurich, ZH, Switzerland	collaborator	LSOS
Sebastian	Winklhofer		Dr	University Hospital Zurich, Radiology	Zurich, ZH, Switzerland	collaborator	LSOS
Roman	Guggenberger		Prof	University Hospital Zurich, Radiology	Zurich, ZH, Switzerland	collaborator	LSOS
Jürg	Hodler		Prof	University Hospital Zurich, Radiology	Zurich, ZH, Switzerland	Chair	Steering Committee, LSOS
Joachim	Oberle		Dr	Cantonal Hospital Winterthur	Winterthur, ZH, Switzerland	collaborator	LSOS
Urs	Schmid		Prof	Triemli Hospital Zurich, Neurosugery	Zurich, ZH, Switzerland	collaborator	LSOS
Min	Kan		Prof	Hirslanden Klinik im Park	Zurich, ZH, Switzerland	collaborator	LSOS
